# Sub-cutaneous Anesthesia with Ether and Oil for Guinea Pigs

**Published:** 1919

**Authors:** 


**Sub-cutaneous Anesthesia with Ether and Oil for Guinea Pigs.** By Captain JAMES T. GWATHMEY, M.C., U. S. A., and Lieutenant SIDNEY W. BLISS, San. Corps, U. S. A. *A Preliminary Report.*

Many animals are required during the war, both in medicine and surgery, to better conserve the soldier's life. Guinea pigs and rabbits are used in the production of reagents for the Wasserman reaction, and rabbits are largely used in the production of diagnostic sera. Dogs and cats help us to decide many problems of the badly wounded soldier, such as shock or the choice of one of several operations.

It is desirable to anesthetize these animals in the most humane, the simplest, quickest and safest way. Theoretically, ether and oil given by the hypodermic needle seem to fulfill these requirements. Anesthesia may be produced quickly and safely, and be made to last thirty minutes, one hour, or an hour and a half to two hours, according to the dosage given.

" Ether was first combined with oil for anesthetic purposes in 1913; since then it has been used in many thousands of cases for colonic anesthesia. Oral analgesia (with ether and oil) for painful dressings has also been used successfully in many cases. " (*British Medical Journal*, March 2, 1918. Sub-Section VII. (b) 163 — 17th International Congress of Medicine, London.)

TECHNIC

In the first series of experiments[1], the hair was removed from the abdomen, the skin was rubbed with alcohol, and the hypodermic

[1] AUTHOR'S NOTE : The primary object of these experiments was to evolve some simple method of analgesia that could be used in the zone of advance. In this we failed.

given under the usual aseptic conditions. A scab was formed which afterwards sloughed away, and many animals died.

The technic was then changed and the hypodermic was given in the side, without removing the hair.

The final technic, which is still not entirely satisfactory, is to give the injection just back of the neck, against the direction of the hairs. Sloughs occurred in all these animals, showing that the amount and kind of oil for this special technic is still to be worked out, and that the method can only be used where complete and final recovery of the animal is not essential. This work is now in progress.

The exact dosage per body weight for a given time was worked as follows :

A predetermined dose of ether was given to two pigs (one weighing 900 grams and one weighing 310 grams). The larger pig was never completely narcotized — never off of its feet — but its movements were inco-ordinated and it was wobbly and weak in the legs. After forty minutes it showed fair control of its limbs, and began to eat, and was fully recovered in an hour. The smaller pig was down in three minutes, was deeply narcotized for one hour — respiration lowered, with abolition of all reflexes — and died at the end of two hours.

The maximum and minimum dose was determined by finding the amount of ether given in each instance cited in grams of body weight.

(1) *The Minimum Dose* : The pig that was only partially anesthetized, weighing 900 grams, was given 4.0 cc. of ether and oil (oil 15 o/o). This may be expressed in the following equation : $\frac{85 \text{ o/o of } 4.0 \text{ cc.}}{9} \frac{3.4}{9} = 0.37$ cc. The minimum dose, therefore, for guinea-pigs is 0.37 cc. of ether per 100 grams of body weight. Several additional experiments verified this as the minimum dose.

(2) *The Maximum Dose* : The pig that died (weighing 310 grams) was given 4.0 of the ether and oil mixture, and the amount of ether may be expressed as follows : $\frac{85 \text{ o/o of } 4.0 \text{ cc.}}{3.1} \frac{3.4}{3.1} = 1.1$ cc., which is equivalent to 1.1 cc. of ether per 100 grams of body weight.

The two following experiments, in addition to the one already cited, verified the maximum dose :

One pig weighing 335 grams was given 4.3 cc. — down in 4 minutes — anesthesia for two hours and 52 minutes ; died

3 hours later. Another weighing the same amount was given the same dosage — down in four minutes — anesthesia for one hour and 26 minutes ; tardy but complete recovery — was too deeply narcotized.

Thus : of three pigs given the maximum dose, two died, and the third was too deeply narcotized at all times. These experiments establish the maximum dose for guinea-pigs.

*The Maximum Safe Dose* : This was obtained by striking an average between the minimum (0.37 cc.) and maximum (1.1 cc.) as follows :

$$\frac{1.1 \text{ plus } 0.37}{2} = \frac{1.47}{2} = 0.74 \text{ cc.}$$

So 0.74 cc. of ether per 100 grams of body weight was found to be the maximum safe dose for surgical anesthesia, lasting from one hour to two hours. Deviations in time may be explained by faulty technic and the vitality of different animals ; for instance, a blind, ill-nourished animal will be much more deeply narcotized than a strong healthy one of the same age.

All animals given this maximum safe dose made a complete recovery from the anesthetic. The following table gives the results of 14 experiments with the maximum safe dose for guinea-pigs.

TABLE I

All animals given a dose corresponding to 0.74 cc. of ether, or 0.87 cc. of the ether-oil mixture, per 100 grams of body weight.

Mixture contained 85 o/o ether and 15 o/o olive oil.

| N° | Weight | Dose Mixture | Time Given. | Down. | On Feet. | Period Anesthesia. | REMARKS |
|---|---|---|---|---|---|---|---|
| 115 | 380 | 3.2 | 4:36 | Never completely anesthetized. | | | Leakage. |
| 132 | 360 | 3.2 | 4:39 | 4:46 | 6:16 | 1 hr. 30 min. | Animal blind leakage. |
| 152 | 360 | 3.2 | 4:42 | 4:48 | 5:57 | 1 hr. 9 " | |
| 117 | 375 | 3.2 | 4:51 | 4:55 | 5:47 | 52 " | |
| 111 | 430 | 3.7 | 5:09 | 5:15 | 5:35 | 20 " | |
| 163 | 440 | 3.8 | 5:12 | 5:15 | 7:35 | 2 hr. 20 " | |
| 187 | 565 | 4.8 | 5:15 | 5:38 | 5:48 | 10 " | |
| 392 | 600 | 5.2 | 5:21 | 5:25 | 5:55 | 30 " | |
| 176 | 330 | 2.8 | 5:24 | 5:27 | 7:45 | 2 hr. 18 " | |
| 133 | 400 | 3.5 | 5:26 | 5:36 | 5:58 | 22 " | |
| 158 | 345 | 3.0 | 5:30 | 5:35 | 7:50 | 2 hr. 15 " | |
| 154 | 355 | 3.0 | 5:34 | 5:38 | 7:42 | 2 hr. 4 " | |
| 156 | 465 | 4.0 | 5:40 | 5:50 | 7:45 | 1 hr. 55 " | |
| 394 | 900 | 7.8 | 5:45 | 5:50 | 7:35 | 1 hr. 45 " | |

An examination of this table shows that eight of the 14 were anesthetized over one hour. Four of these eight were down two hours ; one an hour and a half. Two of the fourteen were down 30 minutes and over ; three of them 10 to 22 minutes, and one not anesthetized at all due to leakage at the point of injection.

A second series of experiments was conducted with the object of ascertaining if a shorter period of anesthesia could be satisfactorily obtained with a smaller dose. This dose is the average between the maximum safe dose (0.74 cc. of ether per 100 grams of body weight) and the minimum dose (0.37 cc. of ether per 100 grams of body weight) and is 0.55 cc. of ether, or 0.65 cc. of the 85 o/o ether-oil mixture, per 100 grams of body weight. This corresponds to one-half the maximum dose. Nine pigs were anesthetized with this amount.

TABLE II

| N° | Weight. | Dose Mixture. | Time Given. | Down. | On Feet. | Anesthesia Period. |
|---|---|---|---|---|---|---|
| 170 | 475 | 3.0 | 1:26 | 1:29 | 2:12 | 43 min. |
| 365 | 480 | 3.0 | 1:31 | Never fully anesthetized. | | |
| 114 | 400 | 2.5 | 1:36 | 1:43 | 3:01 | 1 hr. 18 min. |
| 349 | 440 | 2.8 | 1:40 | 1:43 | 3:00 | 1 hr. 17 » |
| 379 | 300 | 1.9 | 1:44 | 1:45 | died at 2:00 | |
| 105 | 465 | 3.0 | 1:47 | 1:52 | 2:57 | 1 hr. 5 » |
| 366 | 535 | 3.4 | 1:52 | 2:00 | 2:15 | 15 » |
| 350 | 465 | 3.0 | 1:57 | 2:01 | 3:00 | 1 hr. |
| 339 | 355 | 2.2 | 2:10 | 2:14 | 3:38 | 1 hr. 24 » |

It will be seen that five of these nine were anesthetized over one hour, the longest time being one hour 24 minutes ; one 43 minutes; one 15 minutes; one died, and one was not anesthetized.

A third series of experiments was conducted with a still smaller dose — the average between 0.55 cc. and 0.37 cc. of ether, namely, — 0.46 cc. of ether, or 0.54 of the 85 o/o ether-oil mixture, per 100 grams of body weight. Twelve pigs were anesthetized with this amount. The following table gives the result.

Nine of these pigs were anesthetized over 30 minutes, the longest time being 59 minutes, the shortest time being 14 minutes ; two died.

Six more experiments with a still smaller dose were so inconclusive that it was decided the practical minimum dose for anesthesia was represented in the above table.

Table III

| N° | Weight. | Dose Mixture. | Time Given. | Down. | On Feet. | Anesthesia Period. |
|---|---|---|---|---|---|---|
| 360 | 440 | 2.6 | 12:57 | 12:59 | 1:50 | 51 min. |
| 361 | 432 | 2.5 | 1:00 | 1:02 | 1:51 | 49 " |
| 114 | 413 | 2.4 | 1:03 | 1:06 | 2:05 | 59 " |
| 341 | 421 | 2.4 | 1:07 | 1:10 | 1:40 | 30 " |
| 357 | 460 | 2.7 | 1:10 | 1:16 | 1:30 | 14 " |
| 368 | 375 | 2.2 | 1:13 | 1:15 | 1:50 | 35 " |
| 304 | 402 | 2.3 | 1:17 | 1:19 | 1:54 | 35 " |
| 185 | 463 | 2.7 | 1:21 | 1:24 | 1:56 | 32 " |
| 318 | 402 | 2.3 | 1:26 | 1:27 1/2 | | |
| 325 | 440 | 2.6 | 1:30 | 1:33 | | |
| 322 | 385 | 2.2 | 3:30 | 3:33 | 4:10 | 37 " |
| 328 | 365 | 2.1 | 3:33 | 3:36 | 4:13 | 37 " |

Summary

The following doses are the ones that have been determined for guinea-pigs, per 100 grams of body weight (the 85 o/o ether with 15 o/o olive oil being the mixture used in these experiments).

1. *Maximum Safe Dose* : Anesthesia of one to two hours : 0.74 cc. of ether.

Or *0.87 of 85 o/o* ether and 15 o/o oil mixture.

Or *1.00 cc. of 75 o/o* ether and 25 o/o oil mixture.

2. *Medium Dose* : Anesthesia for about one hour : 0.55 cc. of ether.

Or 65 cc. of 85 o/o ether and 15 o/o oil mixture.

Or 0.72 cc. of 75 o/o ether and 25 o/o oil mixture.

3. *Minimum Practical Dose* : Anesthesia of about thirty minutes : 0.46 cc. of ether.

Or 0.54 cc. of 85 o/o ether and 15 o/o oil mixture.

Or 0.60 cc. of 75 o/o ether and 25 o/o oil mixture.

